# Supplementary material for: Predicting categorical and continuous Alzheimer’s disease outcomes from a single MRI scan
Source: Nat Aging. 2026 May 18;6(5):1121–37. doi: 10.1038/s43587-026-01121-2 (PMC13190282; doi:10.1038/s43587-026-01121-2)
Supplement: Supplementary file 1 — Supplementary Figs. 1–4, Methods, Results and Tables 1–9. [file 43587_2026_1121_MOESM1_ESM.pdf]

nature aging

Supplementary information https://doi.org/10.1038/s43587-026-01121-2

Predicting categorical and continuous Alzheimer's disease outcomes from a single MRI scan

In the format provided by the authors and unedited

Section / Item	Title
Supplementary Methods	
S1	Study Data Collection
S1.1	Alzheimer's Disease Neuroimaging Initiative (ADNI)
S1.2	Human Connectome Project (HCP)
S1.3	Dallas Lifespan Brain Study (DLBS)
S2	Data Augmentation
Supplementary Results	
S3	Rigorous 20-iteration Cross Validation of Cognition Task
S4	Model Selection Decisions
S5	Diagnostic Group Comparisons
S6	Comparison of UNet Model Performance with and without Diagnosis Task
S7	Hyper-parameter Tuning
S8	Longitudinal Trajectories
S9	Regional Mean of Occlusion Map
S10	Testing Performance on DLBS Cohort
S11	Diagnosis Confusion Matrix of All Models
Supplementary Tables	
Table S1	Cross-validation results for the best models with 20 iterations of randomized subject order
Table S2	Single-run Validation Results to Aid Model Selection
Table S3	R^2 of model prediction on the set-aside testing set, segregated by diagnostic group
Table S4	Selection of optimal hyperparameters and other model attributes
Table S5	Top 20 regions ranked by average occlusion map (OM) scores derived from the Desikan–Killiany Atlas
Table S6	Modeling testing performance for DLBS cohort set
Table S7	Confusion matrix metrics for each model – ADNI combined set ($n = 1950$)
Table S8	Confusion matrix metrics for each model – ADNI cross-validated set ($n = 1755$)
Table S9	Confusion matrix metrics for each model – ADNI set-aside testing set ($n = 195$)
Supplementary Figures	
Figure S1	ADAS prediction performance of the proposed ensemble model with and without diagnosis task
Figure S2	Model Loss by Training Epochs – Loss convergence for MedicalNet and UNet
Figure S3	Ground truth and predicted longitudinal trajectories of ADAS scores
Figure S4	Confusion matrices illustrating binary classification performance of Models M9 and M10

Supplementary Materials

This Supplementary Materials document has 4 figures and 9 tables, with the corresponding results sections.

1 Supplementary Methods – Study Data Collection

1.1 Alzheimer's Disease Neuroimaging Initiative (ADNI)

The majority of training data were obtained from the Alzheimer's Disease Neuroimaging Initiative (ADNI) database (http://adni.loni.usc.edu), which contains longitudinal cognitive, demographic, fluid, genetic and imaging data on a large sample of AD and control subjects. The study was approved by the Institutional Review Boards at each ADNI site. Informed consent was obtained from all subjects prior to enrollment. All methods were carried out in accordance with relevant guidelines and regulations. For this study we found 2288 subjects in the ADNI 1, 2, 3, and GO metadata dataframe, of which 1950

subjects with (a) current diagnosis status of AD, MCI or Control; and (b) a valid MRI image record for at least one visit. For the longitudinal analysis, we found over 4000 MRI scans for a total of 1950 subjects, who have at least 2 visits recorded. For the longitudinal cognitive score prediction portion, we filtered out those MCI or AD subjects whose longitudinal ADAS-Cog showed improving cognition, which we ascribe to operator noise.

1.2 Human Connectome Project (HCP)

To enlarge the training samples for the segmentation task, we also gathered 1008 healthy young subjects' brain MRIs from Human Connectome Project [1] (https://www.humanconnectome.org/), with age ranging from 20 - 35, processed using the FSL FAST toolset, as our segmentation training enlarge set. HCP is an NIH-awarded data collection research project using state-of-the-art brain imaging technologies; their imaging data are considered to be the standard for neuroimaging in the field of neurodegenerative disease study.

1.3 Dallas Lifespan Brain Study (DLBS)

To further test the model's effectiveness and robustness, we have conducted an external testing dataset using data from the Dallas Lifespan Dataset from the OpenNeuro platform. This is an MRI study on aging subjects unrelated to the ADNI study, including 465 older subjects with MRI, of which 331 subjects have ADAS-Cog scores recorded in wave 2 and wave 3. OpenNeuro is a public, freely accessible and widely used platform; hence its proposed use as an independent cohort will not only strengthen the manuscript, but also provide the wider community an additional resource. This is a sufficiently large sample size that should be more than adequate to support the analysis from the main study. Since this cohort does not include mature Alzheimer patients, it would additionally be quite instructive to assess the applicability of our models trained on the AD spectrum to this new cohort. Please note, although there are other studies with larger samples, they typically lack a comprehensive ADAS-Cog battery, which constituted our main outcome measure.

2 Supplementary Methods - Data Augmentation

To increase the diversity of training data and improve the generalization capability of our deep learning model, we applied elastic transformations during the training phase. Elastic deformation is a non-linear transformation that introduces smooth, spatially coherent displacements to an image, simulating realistic anatomical variability while preserving topology[2]. This method is particularly suitable for medical imaging applications where underlying anatomical structures should not be distorted in unnatural ways [3].

We implemented elastic transformations using the torchio library, a PyTorch-based toolkit for efficient 3D medical image preprocessing and augmentation[4]. Specifically, we used the "torchio.transforms.ElasticDeformation" function, which applies random displacement fields to the image volume by sampling from a Gaussian distribution, followed by B-spline interpolation to generate smooth deformations. The key parameters included a number of control points along all 3 dimensions of the coarse grid to be (3, 3, 3), and the maximum displacement $D_i = 5$ voxels (equal to 5 mms since we are using 1 mm isotropic) along each dimension at each control point to follow a uniform distribution of $d_i \sim \mathcal{U}(0, D_i)$, which were selected to introduce moderate, biologically plausible shape variation without compromising anatomical integrity. The same deformation field was applied simultaneously to both the input image and its corresponding label map to maintain spatial consistency. This augmentation was applied probabilistically during each cross-validated training iteration to encourage the model to learn invariant representations across subtle brain anatomical shifts.

3 Supplementary Results - Rigorous 20-iteration Cross Validation of Cognition task

The results in the main manuscript showed both the cross-validation and set-aside testing results on one split. To impart additional rigor to those results and to assess 95% confidence intervals around

Table 1 Cross validation results for the best models with 20 iterations of randomized subject order. The table compares the performance of different models using various configurations, including the use of segmented volumes as input and different loss functions (MSE and Gamma Loss). For each configuration, it presents the R^2 95% confidence interval on validation sets, average testing R^2, and diagnosis accuracy. Each result therefore corresponds to 200 separate model runs and evaluations. From the CI and range noted in the table, it may be concluded that MedicalNet slightly outperforms the Multi-task UNet for the cognition task, while remaining almost identical for the diagnosis task. Further, the introduction of segmented volumes and Gamma Loss in the training of both models significantly and dramatically improved average testing R^2 and diagnosis accuracy. The boldface elements highlight our best performing models.

	**Segmented Volumes as Input**	**Loss Function**	**R^2 95% Confidence Interval on Validation Sets**	**Average Testing R2**	**Diagnosis Accuracy**
**UNet**	No	MSE	0.69 - 0.74	0.687 (0.635 - 0.702)	0.902
**MedicalNet**	No	MSE	0.71 - 0.74	0.693 (0.649 - 0.710)	0.897
**UNet**	Yes	MSE	0.72 - 0.85	0.784 (0.707 - 0.833)	0.909
**MedicalNet**	Yes	MSE	0.76 - 0.86	0.792 (0.733 - 0.826)	0.925
**UNet**	No	Gamma Loss	0.69 - 0.80	0.775 (0.749 - 0.821)	0.895
**MedicalNet**	No	Gamma Loss	0.69 - 0.78	0.760 (0.691 - 0.822)	0.895
**UNet**	Yes	Gamma Loss	0.76 - 0.88	**0.829 (0.783 - 0.850)**	**0.944**
**MedicalNet**	Yes	Gamma Loss	0.78 - 0.87	**0.846 (0.805 - 0.882)**	**0.942**

those results, we repeated the set-aside validation analysis 20 times, each time fully randomizing the subjects before the splits. We chose for these analyses our two best-performing models, corresponding to the last two rows of Table 1 in the main manuscript. We also repeated these 20-iteration analysis for the other two critical algorithmic choices: use of segmented tissue volumes as input data; and use of conventional versus our custom Gamma loss function. Supplementary Table 1 presents the 20-iteration cross-validation results for the best models, highlighting the performance of 20 × 10 models in particular. It compares different configurations of our neural network architectures: Multi-task UNet or MedicalNet; whether segmented volumes are used as additional input; and the type of loss function applied: MSE or Gamma Loss.

The metrics evaluated include the R^2 confidence interval on validation sets, the average testing R^2, and average diagnosis accuracy. The average testing R^2 is a measure of how well and robust the predicted values correspond with ground truth, with the MedicalNet model with segmented volumes and Gamma Loss achieving the highest average testing R^2 value of 0.846, within a confidence interval of 0.805 to 0.882. The average testing R^2 for the UNet is 0.829, of the same magnitude of significance in the major cognition prediction task.

Overall, the results suggest that ensemble models using segmented volumes and Gamma Loss tend to perform better in terms of R^2 values and diagnostic accuracy. When using MSE as the loss function, MedicalNet50 with segmented volumes achieves the highest R^2 confidence interval (0.76 - 0.86) and the highest diagnosis accuracy (0.925). Multi-task UNet with segmented volumes also performs well with an R^2 confidence interval of 0.72 - 0.85 and a diagnosis accuracy of 0.909. With Gamma Loss, MedicalNet50 with segmented volumes again shows the best performance with an R^2 confidence interval of 0.78 - 0.87, an average testing R^2 of 0.846, and a diagnosis accuracy of 0.942. Multi-task UNet with segmented volumes and Gamma Loss achieves a close second with an R^2 confidence interval of 0.76 - 0.88, an average testing R^2 of 0.829, and a diagnosis accuracy of 0.944.

From the CI and range noted in the table, it may be concluded that MedicalNet slightly outperforms the Multi-task UNet for the cognition task, while remaining almost identical for the diagnosis task. Further, the introduction of segmented volumes and Gamma Loss in the training of both models significantly and dramatically improved average testing R^2 of the cognition task as well as accuracy of the diagnosis task.

4 Supplementary Results - Model Selection Decisions

Supplementary Table 2 shows the preliminary results from the exploration phase of our study, where we simply split the data into 9:1 training and validation sets, and compared multiple modeling options'

performance on the validation set, to instruct our selection of machine learning models. Due to the huge diversity of model options and to economize on overall computational burden, we found it sufficient to assess the suitability of model selections from a single run of training/validation. From the top 4 rows of the table, we can safely conclude that the XGB architecture outperformed the Random Forest and Support Vector Regressor on the baseline cognition task, by achieving R^2 scores over 0.2. The bottom 4 rows, on the other hand, showed that the "3DResNet50" Model in MedicalNet's codebase was superior to the "3DResNet10" Model, compared on both ADNI MRI inputs alone, as well as the ADNI+HCP imaging inputs.

Table 2 Single-run Validation Results To Aid Model Selection

**Model**	**Demog.**	**MRI**	**Val. Dice**	**Val. R^2**	**Val. Acc.**
XGB (Standard MSE)	Yes	No	No	0.24	No
XGB2 (Custom Gamma Loss)	Yes	No	No	0.27	No
Random Forest	Yes	No	No	0.12	No
Support Vector Regressor	Yes	No	No	0.16	No
MedicalNet 3DResnet10	No	ADNI	0.9028	0.63	87.18%
MedicalNet 3DResnet10	No	ADNI + HCP	0.8795	0.64	89.23%
MedicalNet 3DResnet50	No	ADNI	0.9171	0.69	89.74%
MedicalNet 3DResnet50	No	ADNI + HCP	0.9306	0.74	91.79%

5 Supplementary Results - Diagnostic Group Comparisons

All our machine learning models were trained and tested on ADNI subjects regardless of their diagnosis. Here we performed post-hoc analysis on our best-performing models, the 3D UNet and the MedicalNet, both using custom Gamma loss, to assess how well they predicted cognitive scores within different diagnostic groups. Supplementary Table 3 showcases the R^2 of model prediction on the set-aside testing set, categorized by diagnostic group. For the Healthy Control group, with an ADAS-Cog11 score of 12 or less, and consisting of 90 samples, the UNet model reached an R^2 of 0.835, and the MedicalNet R^2 is 0.841. The MCI group, with scores between 12 and 18, includes 47 samples and has lower R^2 values, with UNet at 0.797 and MedicalNet at 0.808. The Dementia group, with scores above 18 and 58 samples, shows R^2 values of 0.822 for UNet and 0.819 for MedicalNet. Thus, model performance is relatively consistent across all groups, with the MCI group having the lowest R^2 values, signifying the fact that this group has the highest clinical heterogeneity within group.

Table 3 R^2 of model prediction on the set-aside testing set, segregated by diagnostic group. While prediction accuracy is comparable across groups, it is lowest for MCI subjects, arguably the most clinically heterogeneous group. The UNet model shown here refers to the model denoted as M9 in main manuscript, and the MedicalNet refers to M10.

**Diagnostic Group**	**ADAS-Cog11 range**	**# samples**	**UNet R^2**	**MedicalNet R^2**
Healthy	ADAS <= 12	90	0.835	0.841
MCI	12 < ADAS <= 18	47	0.797	0.808
Dementia	ADAS > 18	58	0.822	0.819

6 Supplementary Results - Comparison of UNet Model Performance of Cognition with and without Diagnosis Task

Supplementary Figure 1 compares the performance of our proposed ensemble model, with that of the UNet model without the diagnosis task, in predicting ADAS scores. In panel A, we show the prediction performance of the current ensemble model (corresponding to M9: UNet+XGB in the main manuscript). The scatter plot illustrates a strong correspondence between predicted and ground truth

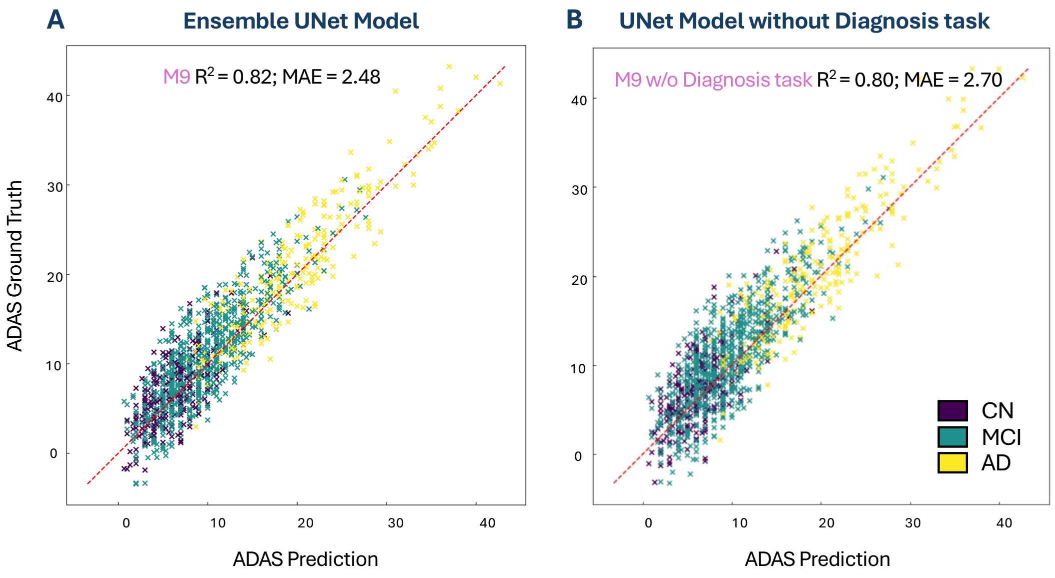

Fig. 1 ADAS prediction performance of the proposed ensemble model with and without diagnosis task. A. Predicted versus ground truth ADAS scores using the ensemble model combining UNet and XGBoost (M9: UNet+XGB), which incorporates both explicit and latent imaging features. **B.** Performance of the UNet model trained without the diagnosis prediction task. The ensemble model achieves higher accuracy ($\mathrm{R}^{\mathbf{2}} = 0.82$, MAE $= 2.48$) compared to the UNet without diagnosis ($\mathrm{R}^{\mathbf{2}} = 0.80$, MAE $= 2.70$), indicating improved prediction of cognitive scores when leveraging the complementary diagnosis classification task.

ADAS scores, with the model achieving an R^2 of 0.82 and a mean absolute error (MAE) of 2.48. In panel B, we present results from the other model, which consists of the same ensemble multi-task UNet pipeline but trained without the diagnosis task. While the latter also shows a strong predictive relationship ($R^2 = 0.80$, MAE $= 2.70$), its performance is slightly lower, suggesting that the integration of an auxiliary diagnosis task enhanced predictive power. These results highlight the benefit of improving cognitive score prediction from structural MRI through adding complementary tasks.

7 Supplementary Results - Hyper-parameter tuning

We assessed model performance as a function of learning rate, the measure of model optimization speed, and found that the optimal performance was achieved at a rate between 10^{-4} to 10^{-5} with a cyclical algorithm. The cyclical learning rates help improve regression accuracy without a need to tune and reduce the number of epochs required [5]. This applies to each model equally.

Weight decay is an L2 regularization technique used in machine learning to prevent overfitting by adding a penalty to the loss function proportional to the magnitude of the model weights. This encourages the optimization process to prefer smaller weights, thereby promoting simpler models that generalize better to unseen data.

Supplementary Figure 2 shows the effect of hyper-parameter selection in the final ensemble model based on the performance of our rigorous cross-validation. The UNet ensemble model reached its optimal validation results on 46 training epochs using the cyclical learning rate algorithm.

Then, we fixed the number of training epochs at 45 and found that a value of 10^{-6} gives the best regularization parameter (i.e. weight decay) for our U-Net-based multi-task model. Similarly, we identified the optimal hyper parameters of MedicalNet as follows: number of epochs $= 44$, weight decay rate of 10^{-6} .

This figure clearly shows that after about 46 epochs the UNet model stops improving and the MedicalNet stops improving after 44 epochs. Evidence for over-fitting is also available on this plot, since it shows that while training loss continues to improve for higher epochs, the validation loss hits a minimum at 40-50 epochs.

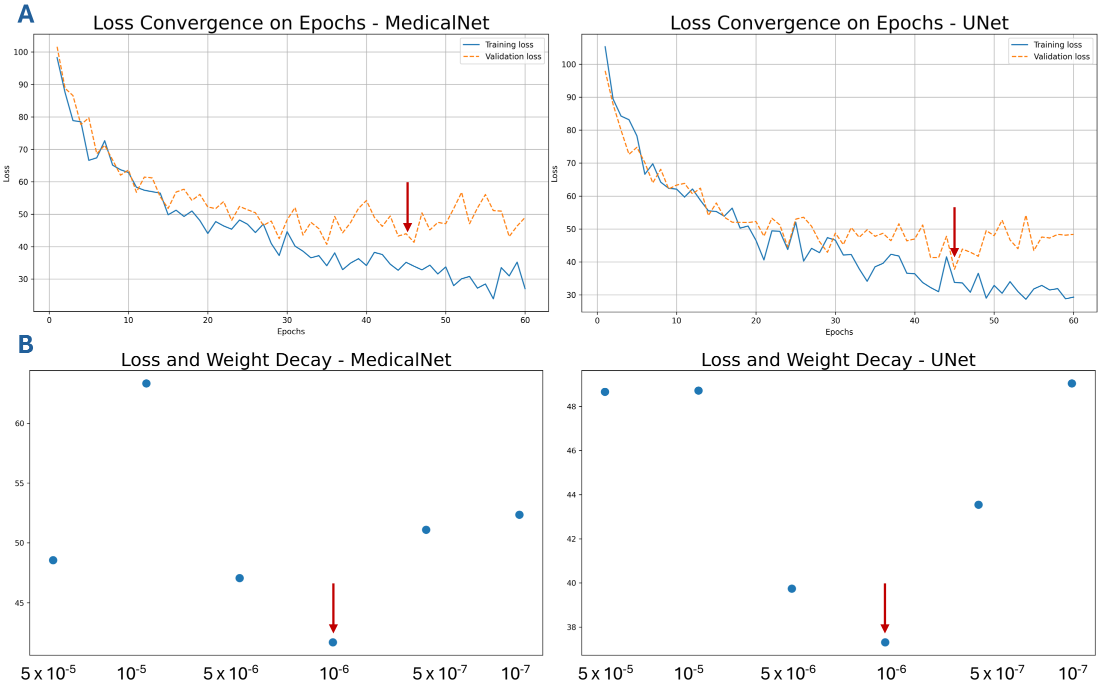

Fig. 2 **A. Model Loss by Training Epochs.** Loss Convergence on Epochs for MedicalNet showing training and validation losses over 50 epochs. The training loss steadily decreases, while the validation loss shows minor fluctuations before stabilizing. At 44 epochs, MedicalNet reaches optimal validation loss, and it takes 46 epochs for the UNet. **B. Model Loss as a function of weight decay rate.** These plots show the best models with different weight decay rates on the x-axis and corresponding losses on the y-axis. The optimal weight decay rates are both 10^{-6}.

Supplementary Table 4 summarizes the optimal model training parameters and other model attributes of the three most pertinent DNN models we implemented. The optimal batch size was limited to 5 and 6, respectively, due to the constraints of the GPU server. Supplementary Figure 2B shows the selection of weight decay rates between the two best ensemble models.

Ensemble UNet has 22.80 million trainable parameters; MedicalNet has 46.21 million total parameters, within which 14.75 million are trainable. The total training time for two best models are 8.12 hours and 7.27 hours from scratch, however, the single sample inference time is shorter, indicating a faster prediction than the average 7-minute-34-second running time of FSL FAST tool, and MedicalNet is slightly faster than the UNet model.

8 Supplementary Results - Longitudinal Trajectories

Table 4 Selection of optimal hyperparameters and other model attributes for three different models: Single-task UNet, Ensemble UNet, and Ensemble MedicalNet. The hyperparameters include batch size, epochs, weight decay, and optimizer. Due to hardware constraints, we fixed the batch size at 6 for Ensemble UNet and 5 for Ensemble MedicalNet. The single-task UNet uses a batch size of 5, 43 epochs, a weight decay of 10^{-6}, and the Adam optimizer. The Ensemble UNet uses a batch size of 6, 46 epochs, a weight decay of 10^{-6}, and the AdamW optimizer. The Ensemble MedicalNet uses a batch size of 5, 44 epochs, a weight decay of 10^{-5}, and the AdamW optimizer. Model attributes are also detailed, indicating the number of parameters (with the number of trainable parameters in parentheses for Ensemble MedicalNet), total training time, and single sample inference time. The Ensemble UNet has fewer total parameters, but more trainable parameters, than the Ensemble MedicalNet. The inference time of both Ensemble models indicate a faster prediction than the average 7-minute-34-second running time of FSL FAST tool, and MedicalNet is faster than the UNet model.

**Hyper parameters**	**Single-task UNet**	**Ensemble UNet**	**Ensemble MedicalNet**
Batch size	5	6	5
Epochs	43	46	44
Dropout	0.2	0.2	0.2
Weight Decay	10^{-6}	10^{-6}	10^{-5}
Optimizer	Adam	AdamW	AdamW
**Model Attributes**			
Number of Parameters (# Trainable)	16.15M	22.80M	46.21M (14.75M)
Total Training Time	6.75 h	8.12 h	7.27h
Single Sample Inference Time	8.7s	10.7s	9.6s

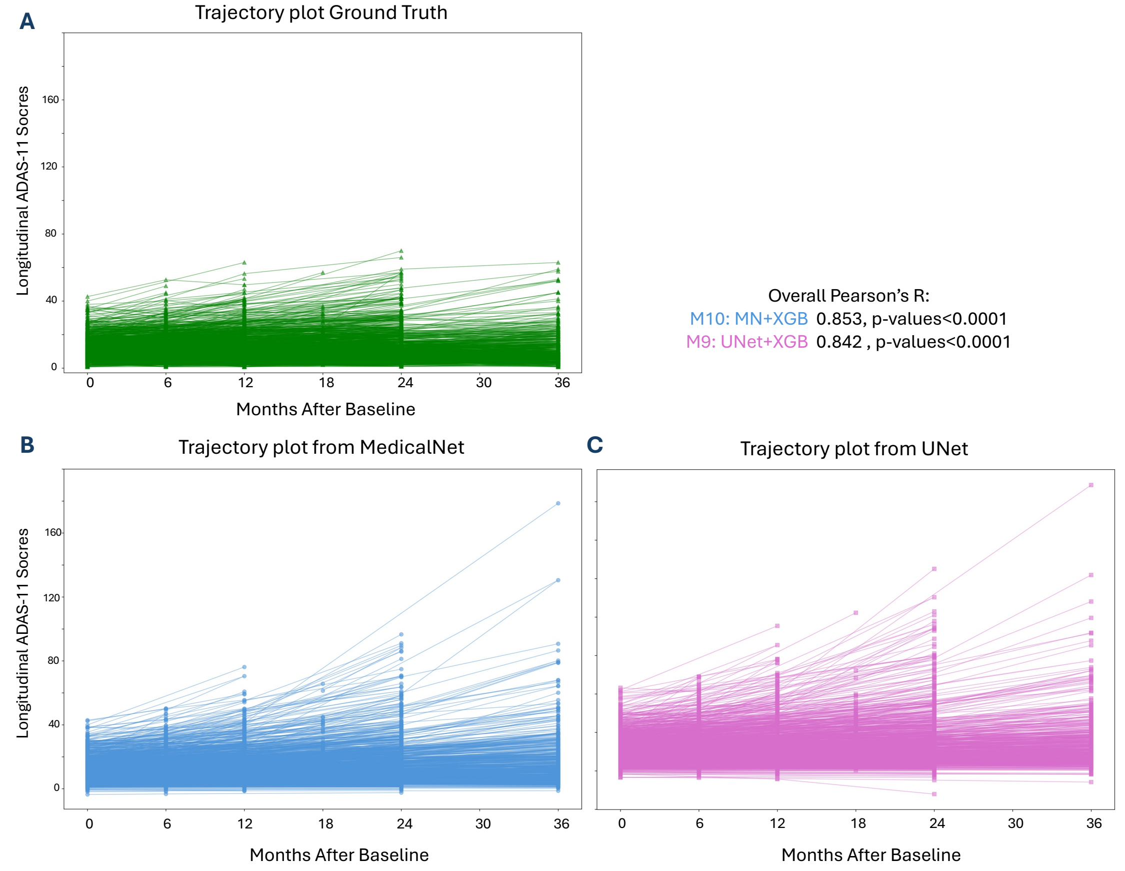

Fig. 3 **A. Ground Truth Trajectory of ADAS Scores.** Here we plot the longitudinal trajectory of the ground truth ADAS-11 scores from the ADNI dataset, each subject connected to a line plot. **B. Predictions from MedicalNet.** This plot shows the trajectory of the predicted cognition scores using the ensemble MedicalNet (model denoted as M10 in main manuscript). These predictions are generated using predicted baseline ADAS with the predicted alphas from the same multi-task training. For the purpose of presentation, all subjects are shown, including the training sets and the testing set. **C. Predictions from UNet.** Similarly, this plot shows the predicted longitudinal trajectory from ensemble model denoted as M9 in main manuscript.

Recall that ADNI contains over 4000 MRI scans on a total of 1950 subjects who have at least 2 visits recorded. To present the overall performance of our top two ensemble models on the cognition task, we plotted the trajectory of longitudinal ADAS-Cog11 scores in Supplementary Figure 3. It is worth noting that we applied the predicted alpha (longitudinal change rate) to the predicted baseline ADAS using the models on the same go.

In these plots, the time points of each subject are connected by a straight line, and the models are colored accordingly. The overall Pearson's R for UNet reaches 0.84, and for MedicalNet it reaches 0.85, both statistically significant with p-values smaller than 0.0001.

9 Supplementary Results - Regional Mean of Occlusion Map

Table 5 Top 20 regions ranked by average occlusion map (OM) scores derived from the Desikan–Killiany (DK) Atlas. These scores quantify the relative importance of each anatomical region to the cognitive predictions made by the U-Net model. Higher average OM scores indicate regions whose occlusion strongly impacts the model's MSE in cognition predictions, thus reflecting their higher contribution to the estimation.

Regions	Average_OM_score
Amygdala_R	0.634146
Paracentral_L	0.622127
Hippocampus_R	0.595255
Parahippocampal_R	0.578119
Hippocampus_L	0.572204
Amygdala_L	0.570938
Paracentral_R	0.563883
Isthmuscingulate_L	0.544527
Fusiform_L	0.543524
Fusiform_R	0.527332
Parahippocampal_L	0.523368
Precuneus_L	0.518676
Isthmuscingulate_R	0.518500
Precuneus_R	0.512507
Accumbens_area_R	0.498265
Posteriorcingulate_L	0.488220
Hypothalamus_R	0.487959
Inferiorparietal_R	0.485582
Caudalanteriorcingulate_L	0.483426
Caudate_R	0.478331

Supplementary Table 5 presents the top 20 anatomical regions identified from occlusion map analysis applied to the U-Net model trained on the AD cohort for cognitive score prediction. The occlusion maps were computed by occluding 3D image patches and assessing the resultant changes in cognition prediction accuracy. Regional averages were subsequently obtained by aggregating voxel-level OM scores according to the Desikan–Killiany (DK) Atlas parcellations. The average occlusion map (OM) scores shown here reflect the relative contribution of each region to model predictions; thus, higher average OM scores indicate areas whose anatomical integrity is critical for accurate cognitive score estimation by the model.

Notably, regions with the highest OM scores include temporal structures such as bilateral hippocampi, amygdalae, and parahippocampal areas — regions consistently implicated in memory processes and known to be early and strongly affected by AD pathology. Additionally, areas within the cingulate cortex, particularly the isthmus cingulate and posterior cingulate cortex, exhibit high scores, underscoring their involvement in the default mode network and their established relevance to cognitive impairment. The prominence of subcortical structures such as the accumbens, caudate, and

hypothalamus further highlights the broad anatomical landscape utilized by the deep learning model in capturing subtle cognitive variations. This region-specific pattern aligns well with established neuropathological evidence and demonstrates that occlusion-based methods provide interpretable insights into the neural correlates of cognitive predictions made by deep learning approaches.

10 Supplementary Results - Testing Performance on DLBS Cohort

Table 6 The modeling testing performance for DLBS cohort set. The relative performance of models M1 to M10 is similar in the DLBS cohort in comparison to the ADNI cohort. Note that the range of ADAS-Cog-11 in DLBS is narrower than that from ADAS, therefore the MAE for DLBS testing are generally lower.

Model	ADNI Testing R^2	ADNI Testing MAE	DLBS Testing R^2	DLBS Testing MAE
M1 XGB (Standard MSE)	0.24	6.69	0.10	5.10
M2 XGB (Gamma Loss)	0.27	6.45	0.12	4.24
M3 Single-Task CNN	0.42	4.18	0.25	3.49
M4 Multitask UNet	0.60	4.92	0.31	3.15
M5 UNet	0.66	4.31	0.39	2.78
M6 MedicalNet	0.58	4.48	0.33	3.11
M7 UNet	0.68	3.97	0.45	2.36
M8 MedicalNet	0.70	2.82	0.44	2.57
M9 Ensemble(UNet +XGB)	0.82	2.48	0.63	1.69
M10 Ensemble (MedicalNet +XGB)	0.80	2.29	0.60	1.88

11 Supplementary Results - Diagnosis Confusion Matrix of All Models

In this section, we report the accuracy of diagnosis task on all of our benchmark models with predicted labels, in three categories. The first one, Table 7, was meant to showcase the overall accuracy on all ADNI subjects, including the cross-validated set of 1755 subjects plus the set-aside set of 195 subjects. Table 8 shows only the cross-validated results on 1755 subjects, and the last Table 9 reports only the set-aside testing results on 175 subjects. Note that we report these predictions only on validated subjects and testing subjects; no training results were reported. For a better visual comparison of the best two models M9 and M10, we report their confusion matrices in Supplementary Figure 4, where subplot A shows the overall results on all 1950 subjects, while subplots B and C show the CV results and set-aside testing results relatively.

From these results, we concluded that the models were quite robust in their relative comparison, especially our two best models M9 and M10. The performance drop between cross-validated set and testing-aside set were not significant.

Table 7 Confusion matrix metrics for each model's diagnosis task on ADNI dataset, cross-validated combined with testing set. (n = 1950)

Model	TP (Non-AD as Positive)	FP	FN	TN (AD as Negative)	Accuracy (%)
M4 Multitask UNet	1425	98	51	376	92.36
M5 UNet	1460	63	90	337	92.15
M6 MedicalNet	1489	34	137	290	91.23
M7 UNet	1465	58	104	323	91.70
M8 MedicalNet	1395	128	27	400	92.05
M9 Ensemble(UNet +XGB)	1450	73	33	394	94.56
M10 Ensemble (MedicalNet +XGB)	1433	90	34	393	93.64

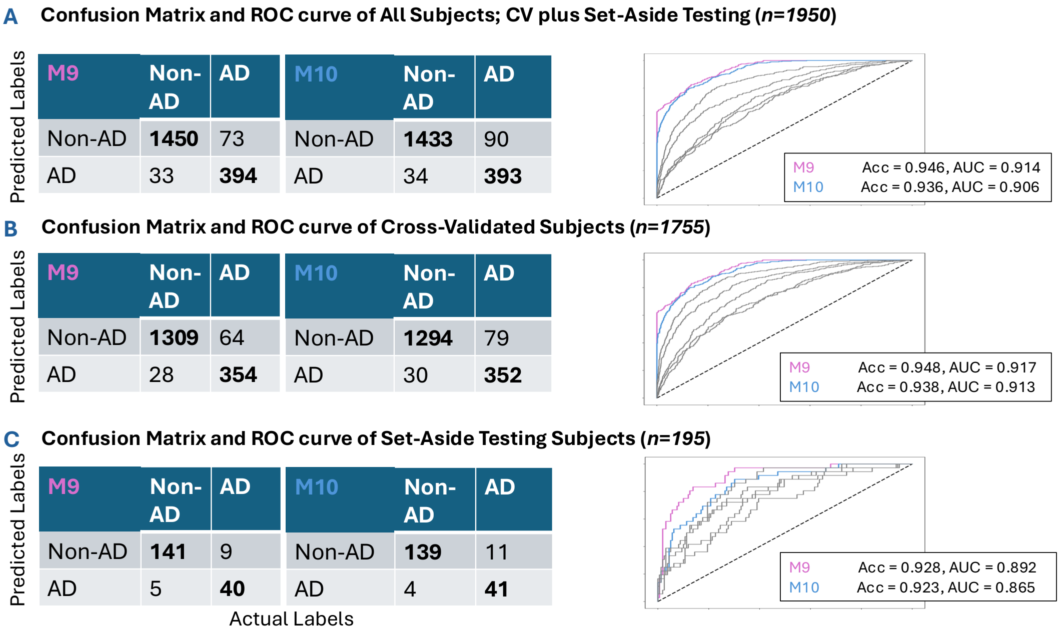

Fig. 4 Confusion matrices illustrating binary classification performance of Models M9 and M10 across all evaluation cohorts. Panels (A–C) show predicted versus actual diagnostic labels for Alzheimer's disease (AD) versus non-AD subjects across three data partitions, along with their ROC curves compared to other benchmark models. We treated Non-AD as Positive, and AD as Negative labels. **A.** The combined sample of cross-validated and set-aside testing subjects (n = 1,950) demonstrates strong discrimination for both models, with Model M9 yielding 1,450 true positives (TP), 394 true negatives (TN), 73 false positives (FP), and 33 false negatives (FN), and Model M10 showing comparable performance with 1,433 TP, 393 TN, 90 FP, and 34 FN. **B.** Confusion matrices for cross-validation folds (n = 1,755) indicate consistent performance stability, where Model M9 produced 1,309 TP, 354 TN, 64 FP, and 28 FN, while Model M10 generated 1,294 TP, 352 TN, 79 FP, and 30 FN. **C.** Results on the held-out testing set (n = 195) confirm generalizability, with Model M9 attaining 141 TP, 40 TN, 9 FP, and 5 FN, and Model M10 yielding 139 TP, 41 TN, 11 FP, and 4 FN. Across cohorts, both models maintained high predictive accuracy with balanced error distributions, supporting their robustness and reliability for AD versus non-AD classification.

Table 8 Confusion matrix metrics for each model's diagnosis task on ADNI dataset, cross-validated set. (n = 1755)

Model	TP (Non-AD as Positive)	FP	FN	TN (AD as Negative)	Accuracy (%)
M4 Multitask UNet	1287	86	41	341	92.76
M5 UNet	1326	47	84	298	92.54
M6 MedicalNet	1352	21	131	251	91.34
M7 UNet	1326	47	97	285	91.79
M8 MedicalNet	1251	122	16	366	92.14
M9 Ensemble (UNet+XGB)	1309	64	28	354	94.76
M10 Ensemble (MedicalNet+XGB)	1294	79	30	352	93.79

Table 9 Confusion matrix metrics for each model's diagnosis task on ADNI dataset, set-aside testing set. (n = 195)

Model	TP (Non-AD as Positive)	FP	FN	TN (AD as Negative)	Accuracy (%)
M4 Multitask UNet	138	12	10	35	88.75
M5 UNet	134	16	6	39	88.72
M6 MedicalNet	137	13	6	39	90.26
M7 UNet	139	11	7	38	90.77
M8 MedicalNet	144	6	11	34	91.28
M9 Ensemble(UNet +XGB)	141	9	5	40	92.82
M10 Ensemble (MedicalNet +XGB)	139	11	4	41	92.30

References

[1] Van Essen, D.C., Ugurbil, K., Auerbach, E., Barch, D., Behrens, T.E., Bucholz, R., Chang, A., Chen, L., Corbetta, M., Curtiss, S.W., *et al.*: The human connectome project: a data acquisition perspective. Neuroimage **62**(4), 2222–2231 (2012)

[2] Simard, P.Y., Steinkraus, D., Platt, J.C.: Best practices for convolutional neural networks applied to visual document analysis. In: Seventh International Conference on Document Analysis and Recognition, 2003. Proceedings., pp. 958–963 (2003). IEEE

[3] Çiçek, Ö., Abdulkadir, A., Lienkamp, S.S., Brox, T., Ronneberger, O.: 3d u-net: learning dense volumetric segmentation from sparse annotation. In: International Conference on Medical Image Computing and Computer-Assisted Intervention, pp. 424–432 (2016). Springer

[4] Paszke, A., Gross, S., Massa, F., Lerer, A., Bradbury, J., Chanan, G., Killeen, T., Lin, Z., Gimelshein, N., Antiga, L., Desmaison, A., Kopf, A., Yang, E., DeVito, Z., Raison, M., Tejani, A., Chilamkurthy, S., Steiner, B., Fang, L., Bai, J., Chintala, S.: Pytorch: An imperative style, high-performance deep learning library. In: Advances in Neural Information Processing Systems, vol. 32. Curran Associates, Inc., ??? (2019). https://papers.nips.cc/paper_files/paper/2019/hash/bdbca288fee7f92f2bfa9f7012727740-Abstract.html

[5] Smith, L.N.: Cyclical Learning Rates for Training Neural Networks (2017). https://arxiv.org/abs/1506.01186
