# Supplementary material for: Predicting categorical and continuous Alzheimer’s disease outcomes from a single MRI scan
Source: Nat Aging. 2026 May 18;6(5):1121–37. doi: 10.1038/s43587-026-01121-2 (PMC13190282; doi:10.1038/s43587-026-01121-2)
Supplement: Supplementary file 2 — Reporting Summary [file 43587_2026_1121_MOESM2_ESM.pdf]

nature portfolio

Corresponding author(s): Daren Ma
Last updated by author(s): Feb 26, 2026

Reporting Summary

Nature Portfolio wishes to improve the reproducibility of the work that we publish. This form provides structure for consistency and transparency in reporting. For further information on Nature Portfolio policies, see our Editorial Policies and the Editorial Policy Checklist.

Statistics

For all statistical analyses, confirm that the following items are present in the figure legend, table legend, main text, or Methods section.

n/a	Confirmed	
☐	☒	The exact sample size (n) for each experimental group/condition, given as a discrete number and unit of measurement
☐	☒	A statement on whether measurements were taken from distinct samples or whether the same sample was measured repeatedly
☐	☒	The statistical test(s) used AND whether they are one- or two-sided<br>*Only common tests should be described solely by name; describe more complex techniques in the Methods section.*
☐	☒	A description of all covariates tested
☐	☒	A description of any assumptions or corrections, such as tests of normality and adjustment for multiple comparisons
☐	☒	A full description of the statistical parameters including central tendency (e.g. means) or other basic estimates (e.g. regression coefficient) AND variation (e.g. standard deviation) or associated estimates of uncertainty (e.g. confidence intervals)
☐	☒	For null hypothesis testing, the test statistic (e.g. F, t, r) with confidence intervals, effect sizes, degrees of freedom and P value noted<br>*Give P values as exact values whenever suitable.*
☒	☐	For Bayesian analysis, information on the choice of priors and Markov chain Monte Carlo settings
☒	☐	For hierarchical and complex designs, identification of the appropriate level for tests and full reporting of outcomes
☐	☒	Estimates of effect sizes (e.g. Cohen's d, Pearson's r), indicating how they were calculated

Our web collection on statistics for biologists contains articles on many of the points above.

Software and code

Policy information about availability of computer code

Data collection	No specific code was used to collect the data. The data were downloaded from public datasets. For the dataset availability, check next section.
Data analysis	Custom code (python version 3.8) for the UNet and Multi-task MedicalNet algorithms will be available online at https://github.com/darenma/MultitaskCognition.

For manuscripts utilizing custom algorithms or software that are central to the research but not yet described in published literature, software must be made available to editors and reviewers. We strongly encourage code deposition in a community repository (e.g. GitHub). See the Nature Portfolio guidelines for submitting code & software for further information.

Data

Policy information about availability of data

All manuscripts must include a data availability statement. This statement should provide the following information, where applicable:

- Accession codes, unique identifiers, or web links for publicly available datasets
- A description of any restrictions on data availability
- For clinical datasets or third party data, please ensure that the statement adheres to our policy

The structural MRI data used in this work are available to researchers via the Human Connectome Project - Young Adults (HCP-YA) data access procedure described at https://www.humanconnectome.org/study/hcp-young-adult/document/extensively-processed-fmri-data-documentation and via the Alzheimer's Disease

Neuroimaging Initiative (ADNI) data access procedure described at http://adni.loni.usc.edu/data-samples/accessdata/.
The ADNI preprocessed MRI data were obtained under account ID daren.ma@ucsf.edu. Source data for the figures will be provided as a Source Data file.

Research involving human participants, their data, or biological material

Policy information about studies with human participants or human data. See also policy information about sex, gender (identity/presentation), and sexual orientation and race, ethnicity and racism.

Reporting on sex and gender	Sex was considered in the study design. We used the "PTGender" column in the ADNI dataset to train the model such that the deep learning structure could capture more biologically relevant underlying features for AD progression.<br>Gender was not considered in this study design.
Reporting on race, ethnicity, or other socially relevant groupings	We didn't use race, ethnicity, or other socially relevant groupings in this study.
Population characteristics	1020 HCP-YA Subjects: 488 Females, 532 Males ; Age: range = 22-37 years, mean = 28.7 years<br>1950 ADNI Subjects: 928 Females, 922 Males ; Age: range = 50-92 years, mean = 72.9 years; ADAS-Cog-11: range = 0.7-42.7, mean=10.3.<br>331 DLBS Subjects: 213 Females, 118 Males; Age: range = 25 - 93 years, mean=62.5, ADAS-Cog-11: range = 0-19.3, mean = 5.2
Recruitment	The human data were obtained from three public datasets: ADNI, HCP-YA, and DLBS.
Ethics oversight	The ADNI study procedures were approved by the institutional review boards of all participating centers as detailed in this document - https://adni.loni.usc.edu/wp-content/uploads/how_to_apply/ADNI_Acknowledgement_List.pdf. Written, informed consent was obtained from all subjects participating in the study according to the Declaration of Helsinki, and the study was approved by the institutional review board at each participating site. The scientific study protocol of the HCP-YA dataset is approved by the Washington University - University of Minnesota Consortium of the Human Connectome Project (WU-Minn HCP),. Written, informed consent was obtained from all subjects participating in the HCP study. The DLBS study procedures were approved by the institutional review boards of all participating centers as detailed in this document - https://openneuro.org/datasets/ds004856/versions/1.2.0. The study was approved by the institutional review board at each participating site.

Note that full information on the approval of the study protocol must also be provided in the manuscript.

Field-specific reporting

Please select the one below that is the best fit for your research. If you are not sure, read the appropriate sections before making your selection.

☒ Life sciences ☐ Behavioural & social sciences ☐ Ecological, evolutionary & environmental sciences

For a reference copy of the document with all sections, see nature.com/documents/nr-reporting-summary-flat.pdf

Life sciences study design

All studies must disclose on these points even when the disclosure is negative.

Sample size	All available HCP-YA and ADNI data that passed quality control procedures as described in data exclusions were retained for this study.<br>The segmentation tasks in this work used sMRI images (n = 1020 + 1950) from unaffected subjects (i.e. those who had no diagnosed or self-reported mental illnesses) from the HCP-YA, and neurodegenerative subjects from the ADNI datasets combined.<br>The baseline cognition regression, and baseline diagnosis classification tasks in this work used sMRI images (n = 1950) from the ADNI repository which satisfied our Alzheimer's disease (AD) progression study criterion.<br>The longitudinal cognition regression task involved 1298 subjects with at least two available visits in the ADNI 1, 2, GO, and 3 dataset, meeting all the study criterion.
Data exclusions	For the HCP-YA samples, we downloaded in total 1200 from the website. After processing using the FSL FAST toolset, there were 1020 scans with successful segmentation output, and we used these samples for the training of the segmentation tasks, along with the valid ADNI MRIs.<br>For this study we found 2288 subjects in the ADNI 1, 2, 3, and GO metadata dataframe, of which 1950 subjects with (a) current diagnosis status of AD, MCI or Control; and (b) a valid MRI image record for at least one visit.<br>For the longitudinal analysis, we found over 4000 MRI scans for a total of 1952 subjects, who have at least 2 visits recorded, and kept 1298 of them. Cognitive batteries are notoriously error-prone and subject to tremendous inter-rater variability [doi:10.1212/01.wnl.0000434309.85312.19]. Therefore, for the longitudinal cognitive score prediction portion, we found it useful to filter out those subjects whose longitudinal ADAS-Cog data can reasonably be deemed error-prone, resulting in a negative progression in terms of cognition scores.<br>For the set-aside testing dataset from DLBS, we recruited all the data that have a baseline ADAS Cog score, in total 331 subjects from DLBS wave2 and wave3.
Replication	A rigorous stratified 9-fold cross-validation procedure was used for nearly all undertaken tasks in this study to ensure reliability of the evaluated performance metrics. The exact same train/validation/test partitions were used across all methods and the performance of all methods was assessed on held-out (unseen) data. All experiments could be successfully validated.

Randomization	Randomization was not performed and is not applicable to our study. We did not collect the MRI data but analyzed public data, and we do not study treatment effects.
Blinding	Blinding was not performed and is not applicable to our study for the exact same reasons. We did not collect the MRI data, but analyzed public data, and we do not study treatment effects.

Reporting for specific materials, systems and methods

We require information from authors about some types of materials, experimental systems and methods used in many studies. Here, indicate whether each material, system or method listed is relevant to your study. If you are not sure if a list item applies to your research, read the appropriate section before selecting a response.

Materials & experimental systems

n/a	Involved in the study
☒	☐ Antibodies
☒	☐ Eukaryotic cell lines
☒	☐ Palaeontology and archaeology
☒	☐ Animals and other organisms
☒	☐ Clinical data
☒	☐ Dual use research of concern
☒	☐ Plants

Methods

n/a	Involved in the study
☒	☐ ChIP-seq
☒	☐ Flow cytometry
☐	☒ MRI-based neuroimaging

Plants

Seed stocks	*Report on the source of all seed stocks or other plant material used. If applicable, state the seed stock centre and catalogue number. If plant specimens were collected from the field, describe the collection location, date and sampling procedures.*
Novel plant genotypes	*Describe the methods by which all novel plant genotypes were produced. This includes those generated by transgenic approaches, gene editing, chemical/radiation-based mutagenesis and hybridization. For transgenic lines, describe the transformation method, the number of independent lines analyzed and the generation upon which experiments were performed. For gene-edited lines, describe the editor used, the endogenous sequence targeted for editing, the targeting guide RNA sequence (if applicable) and how the editor was applied.*
Authentication	*Describe any authentication procedures for each seed stock used or novel genotype generated. Describe any experiments used to assess the effect of a mutation and, where applicable, how potential secondary effects (e.g. second site T-DNA insertions, mosiacism, off-target gene editing) were examined.*

Magnetic resonance imaging

Experimental design

Design type	We used structural MRI scans without any tasks.
Design specifications	MRI data preprocessing details are included in the "Online Methods" Sections
Behavioral performance measures	N/A

Acquisition

Imaging type(s)	T1-weighetd structural
Field strength	3 Tesla
Sequence & imaging parameters	In this study, we included baseline scans of healthy participants from the ADNI-1, ADNI-2, ADNI-Go, and ADNI-3 cohorts. All scans were acquired using an MPRAGE sequence with parameters of TR = 2300/2400–3000 ms, TE ≈ 3 ms, TI = 900–1000 ms, and flip angle = 8°–9°. The images were obtained across multiple sites using scanners from three different manufacturers (GE, Siemens, and Philips). The spatial resolution of ADNI-1 images was 0.9375 mm × 0.9375 mm × 1.2 mm, while that of ADNI-2/Go images was 1 mm × 1 mm × 1.2 mm (Gunter et al., 2009). For the ADNI-3 cohort, the subjects were acquired at 3 T across multiple vendors (GE, Siemens, Philips) using harmonized protocols, with T1-weighted MPRAGE volumes obtained at a resolution of 1 × 1 × 1 mm^3 (Gunter et al., 2017). We did not attempt harmonization between phases for ADNI.
Area of acquisition	We applied the whole brain cortical scans for the imaging models discussed in the paper.

Diffusion MRI ☐ Used ☒ Not used

Preprocessing

Preprocessing software	FSL-FAST and Python Code
Normalization	Non-linear
Normalization template	MNI
Noise and artifact removal	Certain subjects without an accessible ADNI-Cog-11 score or having missing demographic values in the ADNI dataset was removed.
Volume censoring	N/A

Statistical modeling & inference

Model type and settings	Multi-task and multivariate Machine Learning and Deep Learning models were applied to analyze the sMRI data with numerical demographic inputs.
Effect(s) tested	R-squared was computed across the study to test if the model results were reflecting the variance from the data. Diagnosis classification accuracy was evaluated for the classification tasks; Dice's Score was evaluated for the segmentation tasks ;mean squared error, R-squared, and Pearson's correlation (between the observed and true values) metrics were evaluated for the regression tasks.

Specify type of analysis: ☒ Whole brain ☐ ROI-based ☐ Both

Statistic type for inference (See Eklund et al. 2016)	Whole brain
Correction	A permutation feature importance was delivered on the demographic inputs for the two best performing models. No other correction was introduced.

Models & analysis

n/a	Involved in the study
☒	☐ Functional and/or effective connectivity
☒	☐ Graph analysis
☐	☒ Multivariate modeling or predictive analysis

Multivariate modeling and predictive analysis	This study used 2 dimension reduction methods (UNet and MedicalNet), 4 standard machine learning and 6 customized ensemble deep learning models. Diagnosis classification accuracy was evaluated for the classification tasks; Dice's Score was evaluated for the segmentation tasks ;mean squared error, R-squared, and Pearson's correlation (between the observed and true values) metrics were evaluated for the regression tasks.
